# Developmental shape changes in facial morphology: Geometric morphometric analyses based on a prospective, population-based, Chinese cohort in Hong Kong

**DOI:** 10.1371/journal.pone.0218542

**Published:** 2019-06-28

**Authors:** Yi Feng Wen, Hai Ming Wong, Colman P. McGrath

**Affiliations:** 1 Key Laboratory of Shaanxi Province for Craniofacial Precision Medicine Research, College of Stomatology, Xi’an Jiaotong University, Xi’an, China; 2 Clinical Research Center of Shaanxi Province for Dental and Maxillofacial Diseases, College of Stomatology, Xi’an Jiaotong University, Xi’an, China; 3 Paediatric Dentistry, Faculty of Dentistry, The University of Hong Kong, Hong Kong, China; 4 Dental Public Health, Faculty of Dentistry, The University of Hong Kong, Hong Kong, China; Liverpool John Moores University, UNITED KINGDOM

## Abstract

**Background:**

Thorough understanding of developmental changes of human facial shape is lacking. The present study aimed to evaluate developmental shape changes of facial morphology based on a prospective, population-representative, Chinese cohort in Hong Kong.

**Methods:**

A population-representative sample of Chinese in Hong Kong was followed. Serial facial images of over 260 participants were obtained at age 12, 15, and 18 years. Facial landmarks were digitized and the corresponding coordinates were submitted for Generalized Procrustes Analysis. The resultant Procrustes shape coordinates, which captured shape information encoded by the facial landmarks, were then used for statistical shape analyses.

**Results:**

Small but significant developmental changes in mean facial shape were observed (p < 0.0001 for all pairwise comparisons). Significant age-related changes in the magnitude of variance of facial shape were also observed (p < 0.05). Phenotypic growth trajectories representing developmental shape changes were similar in size (p > 0.05) between sexes but differed in direction (p < 0.05) in shape space and trajectory shape (p < 0.05). The magnitude of shape differences between sexes remained constant from 12 to 18 years. Results of frontal facial shape analyses after removing the effect of allometry were similar to results obtained before removal of allometry. For lateral facial configurations, allometric trajectories among the age-by-sex groups were similar in slope (p > 0.05) but varied in directions in the multidimensional shape space.

**Conclusions:**

Our findings suggested significant age-related changes in facial shape and provided a dynamic view of developmental changes in sexual dimorphism of facial shape. Allometry contributed minimally to developmental changes in frontal facial shape. In addition, the allometric trajectories for lateral facial configurations were similar in rate of shape change but differed in their directions in shape space.

## 1 Introduction

Age-related changes in facial characteristics have been traditionally studied based on linear distances, angular measurements, and proportion indices. Disadvantages of this traditional approach to morphometrics are evident [1]. Each measurement alone provides very limited information on the entire craniofacial structure. However, as the number of craniofacial measurement grows, interpretation of these measurements becomes increasingly difficult. Furthermore, the limited number of measurements reported in one study cannot capture all spatial information encoded by the landmark coordinates. This imposes a challenge to graphical illustrations of the craniofacial morphology [2].

In contrast, the geometric morphometric approach to morphometrics, grounded on the statistical theory of shape, captures all geometric information stored in the landmark coordinates and results in a wide range of flexible and powerful statistical procedures for the study of shape [3]. This approach is now being increasingly used in various aspects of facial shape analyses, including facial masculinity [4], facial shape comparison between monozygotic and dizygotic twins [5], and facial variations [6]. In many species of biological organisms, changes in body size are accompanied by changes in body shape. The statistical association between size and shape is termed allometry [7]. Recent studies suggest that allometric variation is significantly associated with variance of facial shape [8].

Geometric morphometrics has been used to investigate the development of craniofacial shape [9]. Comparisons of frontal facial shape of children and adults revealed significant developmental shape changes [9]. However, it remains unknown how such shape differences accumulate throughout the course of development. The study is further limited by its small sample size and exclusive inclusion of male participants. Cephalometric studies suggested that sexual dimorphism of facial shape existed as early as during infancy, which is further accentuated by diverging ontogenetic trajectories during development [10, 11]. In addition to directional differences, there are other trajectory attributes, such as length and shape of the trajectories, that could differ between sexes. Comprehensive geometric morphometric analyses based on serial facial photographs that provide detailed information on developmental changes and sexual dimorphism of facial shape would provide valuable insight into the process of facial development. Age 12 to 18 years represents a period when active facial growth takes place. It is also the period when orthodontic treatment is mostly performed [10]. The present study aimed to evaluate developmental changes in facial shape from age 12 through 15 to 18 years, before and after taking allometry into consideration, based on serial photographic records of a population-representative sample of Chinese in Hong Kong.

## 2 Materials and methods

### 2.1 Study sample

This was a prospective, longitudinal study conducted among a population-representative sample of Chinese in Hong Kong. Details of the study design, sample of the participants, and photographic set-up have been described elsewhere [12]. The sampling frame was all local secondary schools in Hong Kong (by law all children are required to attend secondary school). A random sample of 45 schools (approximately 10% of all local secondary schools) from 18 districts in Hong Kong, special administrative region (SAR), was selected. The secondary schools were the primary sampling unit. Within each school all Form 1 and Form 2 (equivalent to US Grade 6 and 7) students born between April 1st and May 31st, 1997 were invited to participate in the study. At 15 and 18 years of age, the participants were invited for a re-examination. Frontal and lateral facial photographs were taken for each participant during each wave of examination, i.e., at age 12, 15, and 18, respectively. The present study was based on frontal images of 266 (149 females and 117 males) participants and lateral images of 265 (145 females and 120 males) participants from whom complete set of serial photographic records at age 12, 15, and 18 years were available. Parents/primary caregivers gave signed informed consent and participants were asked to provide their assent. The study protocol was approved by the Institutional Review Board of the University of Hong Kong/Hospital Authority Hong Kong West Cluster (IRB reference number: UW 13–584).

### 2.2 Facial landmarks

There were 39 landmarks digitized on frontal images and 14 landmarks digitized on lateral images. Landmark digitization was performed using the software tpsDig2 (http://life.bio.sunysb.edu/morph/soft-dataacq.html). The landmarks (S1 Appendix), selected from those defined Farkas by [13], Fink et al. [14], and Naini [15], were intended to provide coverage of soft tissue facial structures. Facial landmarks digitized in one of our earlier publications [12] were directly used for the present study. Several additional facial landmarks were also digitized for this study. Schematic illustrations of facial landmarks used in this study are provided in S1 and S2 Figs.

The landmarks were digitized by one trained investigator (YFW) and were checked for accuracy by another (HMW). For assessment of measurement error in landmark digitization, frontal and lateral photos of 50 randomly selected participants (25 females and 25 males) were digitized four times by one investigator (YFW) as described elsewhere [12]. All datasets for this study are included in the manuscript and the supplementary files (S1–S4 Data).

### 2.3 Generalized procrustes analyses

Landmark data from lateral images were directly used for geometric morphometric analyses. Since the human face demonstrates object symmetry, landmarks digitized on frontal facial images were symmetrized by calculating the mean of the original coordinates and their reflected relabeled copies. Facial landmarks were then superimposed using Generalized Procrustes Analysis (GPA), which served to remove nonshape information including location, orientation, and centroid size (CS) of the configurations. The resultant Procrustes shape coordinates and CS were used for subsequent statistical analyses. These procedures were implemented using the “bilat.symmetry” function for frontal facial configurations and the “gpagen” function for lateral facial configurations from the geomorph package [16] in R software.

### 2.4 Interaction between CS and age-by-sex groups

One-way repeated measures analysis of variance (ANOVA) with pairwise comparisons was used to examine changes of *In*(*CS*) with age. To determine if the age-by-sex groups (three age levels by two sexes) differed in patterns of allometry, permutational multivariate analysis of covariance (MANCOVA) was performed using the following formula:

Facialshape∼In(CS)×group,

where *Facial shape* contains Procrustes shape coordinates of frontal or lateral facial landmark configurations, *In*(*CS*) denotes natural logarithm of CS, and *group* indicates the six age-by-sex groups. A total of 10000 permuted samples were derived for determination of statistical significance. The ‘adonis’ function in the vegan package [17] was used for the MANCOVA analyses.

Results from MANCOVA (Table 1) showed that the interaction between age-by-sex groups and *In*(*CS*) was nonsignificant for frontal facial configurations (p = 0.2949), hence the allometric effect was consistent among the age-by-sex groups for frontal facial configurations. This allowed for removal of the allometric effect from Procrustes shape coordinates by standardizing *In*(*CS*) at a fixed value [18]. In contrast, there was significant interaction between lateral facial shape and *In*(*CS*) (p = 0.0002), which indicated that the size-standardization approach for removal of allometric effect was not appropriate because the magnitude of shape difference among the age-by-sex groups depended on the value of *In*(*CS*) selected for standardization.

**Table 1:** Statistical significance of the interaction between *In*(*CS*) and age-by-sex groups.

MANCOVA Model	Frontal images			Lateral images		
	partial *R*^2^	*p*-value		partial *R*^2^	*p*-value	
*In*(*CS*)	0.0194	0.0001	***	0.0220	0.0001	***
age×sex	0.0656	0.0001	***	0.0546	0.0001	***
*In*(*CS*)×(age×sex)	0.0110	0.2949		0.0131	0.0002	***

***p< 0.001.

Partial *R*^2^ = coefficient of partial determination.

### 2.5 Developmental shape changes of frontal facial morphology

Developmental shape changes of frontal facial morphology were evaluated, as described in section 2.5.1 to 2.5.4, using Procrustes shape coordinates and size-corrected Procrustes shape coordinates separately, which corresponded to shape changes before and after removal of the allometric effect, respectively. The size-corrected Procrustes shape coordinates were estimated as residuals from a Procrustes regression model in which shape data for frontal facial morphology across all age levels were modeled as a function of *In*(*CS*), when *In*(*CS*) was fixed at its grand mean (mean of all *In*(*CS*)s collapsing across all age levels).

#### 2.5.1 Developmental changes in mean facial shape

To determine whether facial shape changed as a function of age, permutational multivariate analysis of variance (MANOVA) was performed using the formula:

Facialshape∼Age+Gender+Age×Gender,

where *Facial shape* contains Procrustes shape coordinates or size-corrected Procrustes shape coordinates of frontal facial configurations over all three age levels, *Age* is a categorical variable of three levels, indicating age 12, 15, and 18 years, and *Gender* is a categorical variable of two levels. Since longitudinal data have an inherent hierarchical structure (age levels were nested within participants), permutations were performed across age levels but were restricted within each participant, as recommended by Anderson and Braak [19]. The “adonis” function in the vegan package was used for the MANOVA procedures.

Coefficient of partial determination (*partial R*^2^) of a term in the above model was calculated using the following formula [20]:

$$partial\;R^{2}=\frac{Residual\;SS_{reduced}-Residual\;SS_{full}}{Residual\;SS_{reduced}},$$

where *Residual SS*_*reduced*_ is the residual sum of square of the reduced model that lacks the term and *Residual SS*_*full*_ is the residual sum of square of the fuller model that contains the term.

Subsequently, statistical significance of shape difference between any two age levels was determined from the following model:

Facialshape′∼Age′,

where *Facial shape*′ contains Procrustes shape coordinates for the two age levels under comparison and *Age*′ is a group indicator for the two age levels. This model was applied separately for each sex. The amount of shape difference between two age levels was represented by partial Procrustes distance (PD).

Principal component analysis (PCA) is an ordination method routinely used in geometric morphometric analyses to visualize shape variation among individuals. To facilitate visualization of frontal facial shape development, PCA was performed based on covariance matrix of the Procrustes shape coordinates through the ‘plotTangentSpace’ function in the geomorph package. Principal component (PC) plots were then constructed using the R package ggplot2 [21] to represent each age-by-sex group’s mean score along PC1 and PC2. Thin-plate spline deformation grids (TPS grids) were plotted to allow for visualization of changes in facial shape along the PCs. In addition, Procrustes shape coordinates for mean shape of each age-by-sex group were computed through the ‘mshape’ function in the geomorph package and were plotted using ggplot2. Superimpositions of the resultant plots were then performed by sex, which allowed for direct visualization of developmental shape changes of frontal facial morphology.

#### 2.5.2 Developmental changes in variance of facial shape

Variance of the facial shape was estimated for each age-by-sex group using the following formula [22]:

$$Variance=\frac{\sum_{i=1}^{n}d_{i}^{2}}{n-1},$$

where $d_{i}^{2}$ is the squared PD of the *i*^*th*^ participant’s facial shape from the group’s mean shape and *n* is the group sample size. Pairwise differences in variance of facial shape across age levels were then calculated and the p values were derived from the number of times that the observed changes in variance were exceeded by the 10000 permuted samples. The analyses were performed using DisparityBox8 module nested in the PCAGen8 software (available at: http://www3.canisius.edu/~sheets/IMP%208.htm).

#### 2.5.3 Comparisons of phenotypic growth trajectories between sexes

Phenotypic growth trajectories are characterized by trajectory size, which quantifies the amount of shape changes from 12 to 18 years; trajectory orientation, which describes the direction of shape change in the multidimensional shape space; and trajectory shape, which measures the shape of the path along which developmental shape changes take place [23]. Sexual dimorphism in characteristics of phenotypic growth trajectories were analyzed using the “trajectory.analysis” function in the geomorph package. Statistical significance was determined by comparing the observed sex difference against the sampling distribution generated by 5000 residual randomization permutations.

#### 2.5.4 Developmental changes in sexual dimorphism of facial shape

The magnitude of sexual dimorphism of facial shape was quantified with PD. To determine if the magnitude of sexual dimorphism changed as a function of age, PD was compared pairwise among age levels and the results were described by 95% confidence intervals derived from 4900 bootstrapped samples. Significant developmental changes in sexual dimorphism were declared if the intervals did not transgress the zero point. The analyses were performed in the TwoGroup8 software (available at: http://www3.canisius.edu/~sheets/IMP%208.htm)

### 2.6 Developmental shape changes of lateral facial morphology

Evaluation of developmental shape changes of lateral facial morphology without removal of the allometric effect was performed in the same way as described in section 2.5.1 to 2.5.4 for analyses of frontal facial morphology. However, when allometric effect was to be removed for evaluation of changes in lateral facial shape, the size-standardization approach to remove allometric effect was no longer appropriate due to significant interaction between *In*(*CS*) and the age-by-sex groups (Table 1, p = 0.0002). As a result, a different set of statistical procedures was performed, as described in section 2.6.1 to 2.6.4, for shape analyses removing the effect of allometry.

#### 2.6.1 Patterns of allometry in each age-by-sex group

Since biological variations in CS are multiplicative by nature [24], log-transformed CS was used for investigation of allometry, as has been indicated and used in earlier studies [25–27]. Therefore, multivariate regression of lateral shape on *In*(*CS*) was implemented for each age-by-sex group to examine pattern of allometry in each age-by-sex group. The analyses were performed using the procD.lm function in the geomorph package. The p values were obtained from 10000 random permutations.

To visualize patterns of allometry for the age-by-sex groups, mean facial shape at each group’s smallest and largest value of *In*(*CS*) were predicted from the group-specific regression models. Residuals of the regression models were then added back to the predicted mean facial shapes. This resulted in 12 categories of Procrustes shape coordinates where each category contained predicted shape variables at the smallest or largest *In*(*CS*) of a particular age-by-sex group. All predicted shape variables were then jointly submitted to PCA. PC plot was then used to represent each of the 12 categories’ mean PC 1 and PC 2 score. Points representing predicted mean shapes at each age-by-sex group’s smallest and largest *In*(*CS*) were connected by a straight line so that patterns of allometry could be visualized along the first two PCs. A similar approach has been used elsewhere [28].

#### 2.6.2 Visualizing developmental changes of lateral facial shape at different levels of *In*(*CS*)

Since the age-by-sex groups differ significantly with respect to allometry, direct comparison of later facial shape at different age levels is complicated by the effect of *In*(*CS*). Therefore, developmental shape changes were visualized at each group’s smallest/largest *In*(*CS*). All comparisons were restricted within sex.

#### 2.6.3 Rate of change in lateral facial shape with *In*(*CS*) among the age-by-sex groups

To determine whether rate change in lateral facial shape with *In*(*CS*) differed among the age-by-sex groups, pairwise differences in the amount of shape change (measured in PD) per unit change of *In*(*CS*) were tested using the “advanced.procD.lm” function in the geomorph package. The p values were obtained from 10000 residual randomization permutations [29].

#### 2.6.4 Direction of change in lateral facial shape with *In*(*CS*) among the age-by-sex groups

To determine whether the age-by-sex groups’ allometric trajectories differed in orientation in the multidimensional shape space, angles between the trajectories were calculated using the “advanced.procD.lm” function in the geomorph package. The p values were obtained from 10000 residual randomization permutations [29].

### 2.7 Quantification of measurement error

Measurement error resulting from landmark digitization was quantified using the %*ME* measure introduced by Yezerinac et al [30] based on permutational MANOVA model. The analysis was implemented with the “procD.lm” function in the geomorph package. Repeatability [31], which reflects the percent of total variation associated with among-individual variation, is then determined through the formula: *Repeatability* = 1−%*ME*.

## 3 Results

### 3.1 Quantification of measurement error

Permutational MANOVA indicated that there was no significant difference among the four replication sessions in mean shapes of the frontal (p = 0.1374) or lateral (p = 0.3142) facial landmark configurations. The %*ME* was 0.82% and 0.86% for digitization of landmarks on frontal and lateral images, respectively. Accordingly, the repeatability of landmark digitization was 99.18% for frontal images and was 99.14% for lateral images.

### 3.2 Growth changes in CS

Size of facial configurations were described using *In*(*CS*) (Table 2). In frontal view, *In*(*CS*) increased steadily from 12 to 15 years (females: p = 0.006, males: p < 0.001) and from 15 to 18 years (females: p < 0.001, males: p < 0.001) for both sexes. The *In*(*CS*) in lateral view decreased from 12 to 15 years (p = 0.024) but increased from 15 to 18 years (p < 0.001) for females. For males in lateral view, *In*(*CS*) was stable from 12 to 15 years (p = 1.0000) and increased from 15 to 18 years (p < 0.001). Distributions of *In*(*CS*) were described using boxplot (S3 Fig).

**Table 2:** Descriptive and analytical statistics for *In*(*CS*).

		Frontal images				Lateral images			
		Mean	SD	Overall sig.		Mean	SD	Overall sig.	
Females				<0.001	***			0.013	*
	12 years	3.41	0.04			2.92	0.07		
	15 years	3.42	0.04			2.91	0.04		
	18 years	3.43	0.03			2.92	0.04		
Males				<0.001	***			0.004	**
	12 years	3.47	0.05			2.98	0.07		
	15 years	3.47	0.04			2.98	0.04		
	18 years	3.49	0.04			2.99	0.04		

***p<0.001

**p<0.01

*p<0.05.

Overall sig. = overall significance; SD = standard deviation.

### 3.3 Developmental shape changes of frontal facial morphology

#### 3.3.1 Developmental changes in mean facial shape

Permutational MANOVA (S2 Appendix) revealed that there was significant main effect of age (p = 0.0001) and sex (p = 0.0001) on frontal facial morphology. Because of the significant interaction between age and sex (p = 0.0003), pairwise comparisons of facial shapes among age levels were performed separately by sex (Table 3). Significant shape difference was noted for each comparison performed (p = 0.0001) both before and after removal of allometric effect. It was noteworthy that the magnitude of shape change from 12 to 18 years, both before and after removal of allometric effect, was evidently smaller than the sum of changes from 12 to 15 years and from 15 to 18 years. This suggested that the direction of developmental changes of frontal facial shape was not consistent over the entire observation period. In addition, the magnitude of developmental changes in mean frontal facial shape was very similar before and after removal of allometry.

**Table 3:** Pairwise differences in mean shapes among age levels.

			12 years vs 15 years			15 years vs 18 years			12 years vs 18 years		
			PD	*p*-value		PD	*p*-value		PD	*p*-value	
**Before CS correction**											
	Frontal images										
		Females	0.0125	0.0001	***	0.0201	0.0001	***	0.0170	0.0001	***
		Males	0.0131	0.0001	***	0.0210	0.0001	***	0.0244	0.0001	***
	Lateral images										
		Females	0.0108	0.0001	***	0.0196	0.0001	***	0.0198	0.0001	***
		Males	0.0165	0.0001	***	0.0133	0.0001	***	0.0228	0.0001	***
**After CS correction**											
	Frontal images										
		Females	0.0125	0.0001	***	0.0192	0.0001	***	0.0156	0.0001	***
		Males	0.0130	0.0001	***	0.0202	0.0001	***	0.0233	0.0001	***

***p<0.001.

PD = partial Procrustes Distance.

**Fig 1:**
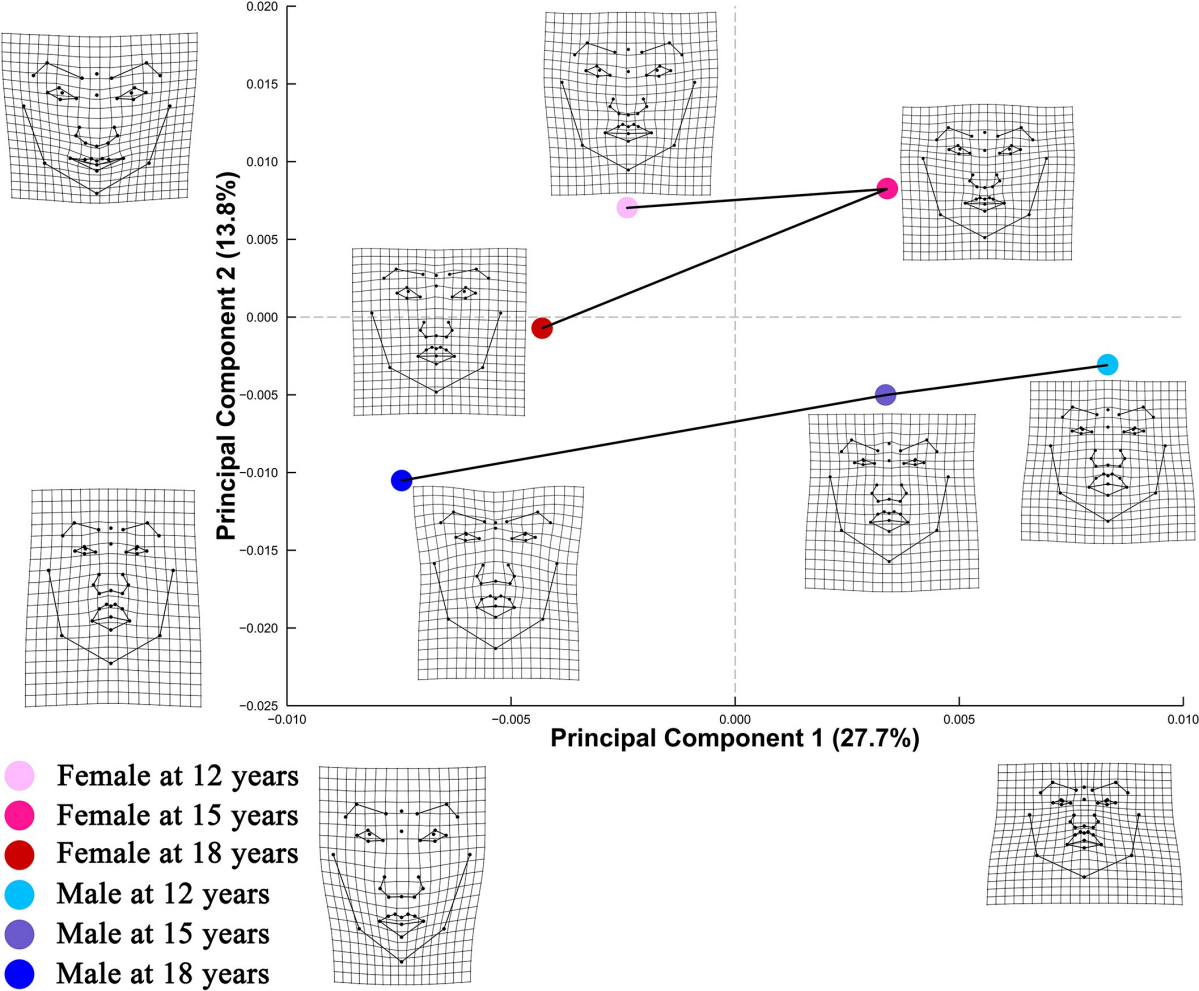
PC plot of Procrustes shape coordinates for frontal facial landmark configurations. Points represent mean PC score for the age-by-sex groups along the first two PCs. Black lines are two dimensional representations of the phenotypic growth trajectories that exist in the multidimensional shape space. The relative amount of shape variation explained by the first two PCs are shown in percentage. TPS grids along the axes illustrate shape difference between configurations with low/high PC scores and the grand mean shape, with ×2.5 magnification of the real shape difference. Mean shape for each age×sex group is illustrated relative to the grand mean shape using TPS grids next to the corresponding point, with ×4 magnification.

**Fig 2:**
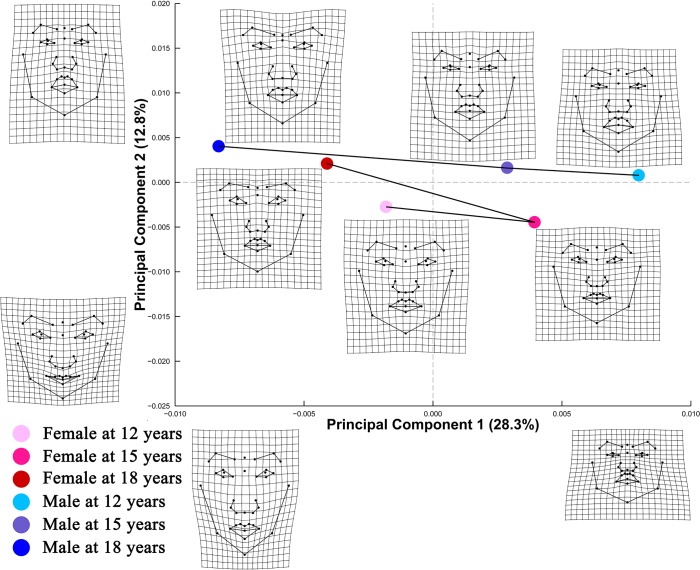
PC plot of Procrustes shape coordinates for frontal facial landmark configurations after size standardization. Points represent mean PC score for the age-by-sex groups along the first two PCs after size standardization. Black lines are two dimensional representations of the phenotypic growth trajectories that exist in the multidimensional shape space. The relative amount of shape variation explained by the first two PCs are shown in percentage. TPS grids along the axes illustrate shape difference between configurations with low/high PC scores and the grand mean shape, with ×2.5 magnification of the real shape difference. Mean shape for each age×sex group is illustrated relative to the grand mean shape using TPS grids next to the corresponding point, with × 4 magnification.

PCA of Procrustes shape coordinates of frontal facial landmark configurations (Fig 1) revealed that PC1 accounted for 27.7% of shape variation, distinguishing facial shapes that differ in spatial arrangement of landmarks for the middle and lower facial area. PC2 accounted for 13.8% of shape variation, addressing different spatial distributions of lower facial landmarks, particularly those governing the relative thickness of upper and lower lips. PC plot from PCA of size-corrected Procrustes shape coordinates demonstrated similar shape changes along PC 1 and PC 2 (Fig 2).

**Fig 3:**
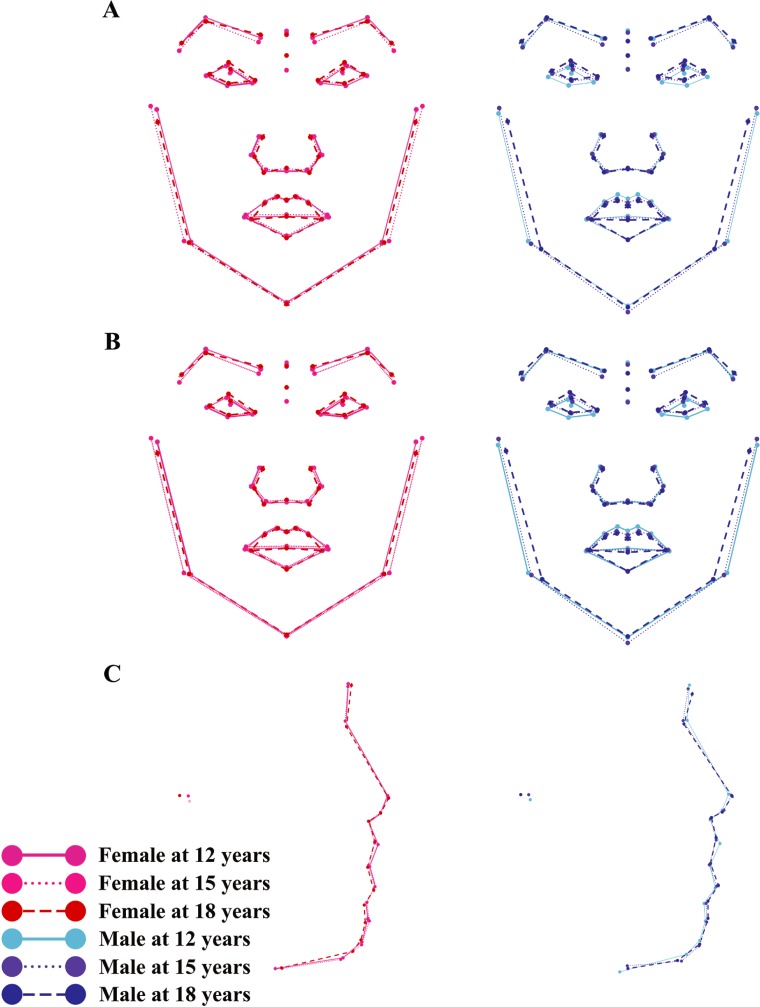
Visualization of developmental shape changes of facial morphology. Facial shapes at three age levels are superimposed to facilitate visualization, with ×4 magnification. (A) Shape development of frontal facial morphology before removing allometry; (B) shape development of frontal facial morphology after removing allometry; (C) shape development of lateral facial morphology before removing allometry.

Phenotypic growth trajectories along the first two PCs showed that developmental changes of facial shape did not take place in a linear fashion (Figs 1 and 2). Superimposed facial shapes (Fig 3A and 3B) revealed that for several landmarks, the direction of displacement from 15 to 18 years was opposite to the direction of displacement from 12 to 15 years.

#### 3.3.2 Developmental changes in variance of facial shape

For females (Table 4), variance of frontal facial configurations decreased 16.0% from 12 to 15 years (p = 0.0068) and 20.0% from 12 to 18 years (p = 0.0002). For males (Table 4), variance of frontal facial shape remained constant (p > 0.05) during the observation period. Similar changes in variance of facial shape were observed after removal of allometry.

**Table 4:** Shape variance at each age level and pairwise comparisons among age levels.

				Variance at each age level			Pairwise comparisons (*p*-value)				
				12 years	15 years	18 years	12 vs 15 years		15 vs 18 years	12 vs 18 years	
**Before CS correction**											
	Frontal images										
		Females		0.0025	0.0021	0.0020	0.0068	**	0.3848	0.0002	***
		Males		0.0025	0.0024	0.0024	0.6588		0.8700	0.5914	
	Lateral images										
		Females		0.0044	0.0039	0.0038	0.0894		0.5899	0.0193	*
		Males		0.0047	0.0044	0.0041	0.2327		0.2841	0.0257	*
**After CS correction**											
	Frontal images										
		Females		0.0025	0.0021	0.0020	0.0065	**	0.4907	0.0005	***
		Males		0.0025	0.0024	0.0024	0.6978		0.9021	0.6241	

***p<0.001

**p<0.01

*p<0.05.

#### 3.3.3 Comparisons of phenotypic growth trajectories between sexes

Table 5 revealed that the direction of developmental changes in frontal facial shape differed between sexes (*θ* = 41.3996, *p* value = 0.0038). In addition, females and males differed significantly in shapes of the phenotypic growth trajectories (*D*_*shape*_ = 0.2423, *p* value = 0.0218). In contrast, the total amount of shape change was similar between sexes (*MD* = 0.0014, *p* value = 0.6742). Removal of allometry had no substantial impact on sexual dimorphism of phenotypic growth trajectories.

**Table 5:** Differences in phenotypic trajectory attributes between sexes.

		Size			Orientation				Shape			
		*MD*	*Z*	*p*-value	*θ*	*Z*	*p*-value		*D*_*shape*_	*Z*	*p*-value	
**Before CS correction**												
	Frontal images	0.0014	0.4086	0.6742	41.3996	1.8542	0.0038	**	0.2423	1.9547	0.0218	*
	Lateral images	-0.0006	0.1179	0.9057	93.1695	1.7875	0.0406	*	0.3876	1.7357	0.0452	*
**After CS correction**												
	Frontal images	0.0015	0.4127	0.6681	44.4152	1.8248	0.0048	**	0.2629	1.9783	0.0194	*

**p< 0.01

*p< 0.05.

*MD* = mean difference in magnitude of phenotypic change (magnitude of mean change for females were subtracted from mean change for males) measured in Euclidean distances; *θ =* mean difference in general direction of phenotypic change measured in degrees; *D*_*shape*_ = mean difference in shape of phenotypic growth trajectory measured in Euclidean distances; *Z* = effect size.

#### 3.3.4 Developmental changes in sexual dimorphism of facial shape

Analyses based on Procrustes shape coordinates characterizing frontal facial morphology before and after removal of allometry suggested that the amount of sexual dimorphism in facial shape remained stable from age 12 through 15 to 18 years, as shown by the pairwise 95% confidence intervals that all included the zero point (Table 6).

**Table 6:** Shape difference between sexes at each age level and pairwise comparisons among age levels.

			12 years	15 years	18 years
**Before CS correction**					
	Frontal				
		12 years	0.0215	(-0.0064, 0.0028)	(-0.0082, 0.0012)
		15 years		0.0197	(-0.0055, 0.0025)
		18 years			0.0181
	Lateral				
		12 years	0.0288	(-0.0099, 0.0039)	(-0.0089, 0.0056)
		15 years		0.0257	(-0.0056, 0.0086)
		18 years			0.0271
**After CS correction**					
	Frontal				
		12 years	0.0173	(-0.0077, 0.0015)	(-0.0085, 0.0015)
		15 years		0.0142	(-0.0046, 0.0038)
		18 years			0.0137

Values along the diagonal indicate shape difference between sexes at each age level; above diagonal are 95% confidence intervals for pairwise comparisons of sexual dimorphism among age levels. The pairwise comparisons were performed by subtracting sexual dimorphism at the younger age from the difference at the older age.

### 3.4 Developmental shape changes of lateral facial morphology

#### 3.4.1 Developmental shape changes without removal of allometric effect

*Developmental changes in mean facial shape*: Permutational MANOVA (S2 Appendix) revealed that there was a significant main effect of age (p = 0.0001) and sex (p = 0.0001) on lateral facial morphology. Because of the significant interaction between age and sex (p = 0.0001), pairwise comparisons of facial shapes among age levels were performed separately by sex (Table 3). Significant shape difference was noted for each comparison performed (p = 0.0001). It was noteworthy that the magnitude of shape change from 12 to 18 years was evidently smaller than the sum of changes from 12 to 15 years and from 15 to 18 years. This suggested that the direction of developmental changes of lateral facial shape was not consistent over the entire observation period.

**Fig 4:**
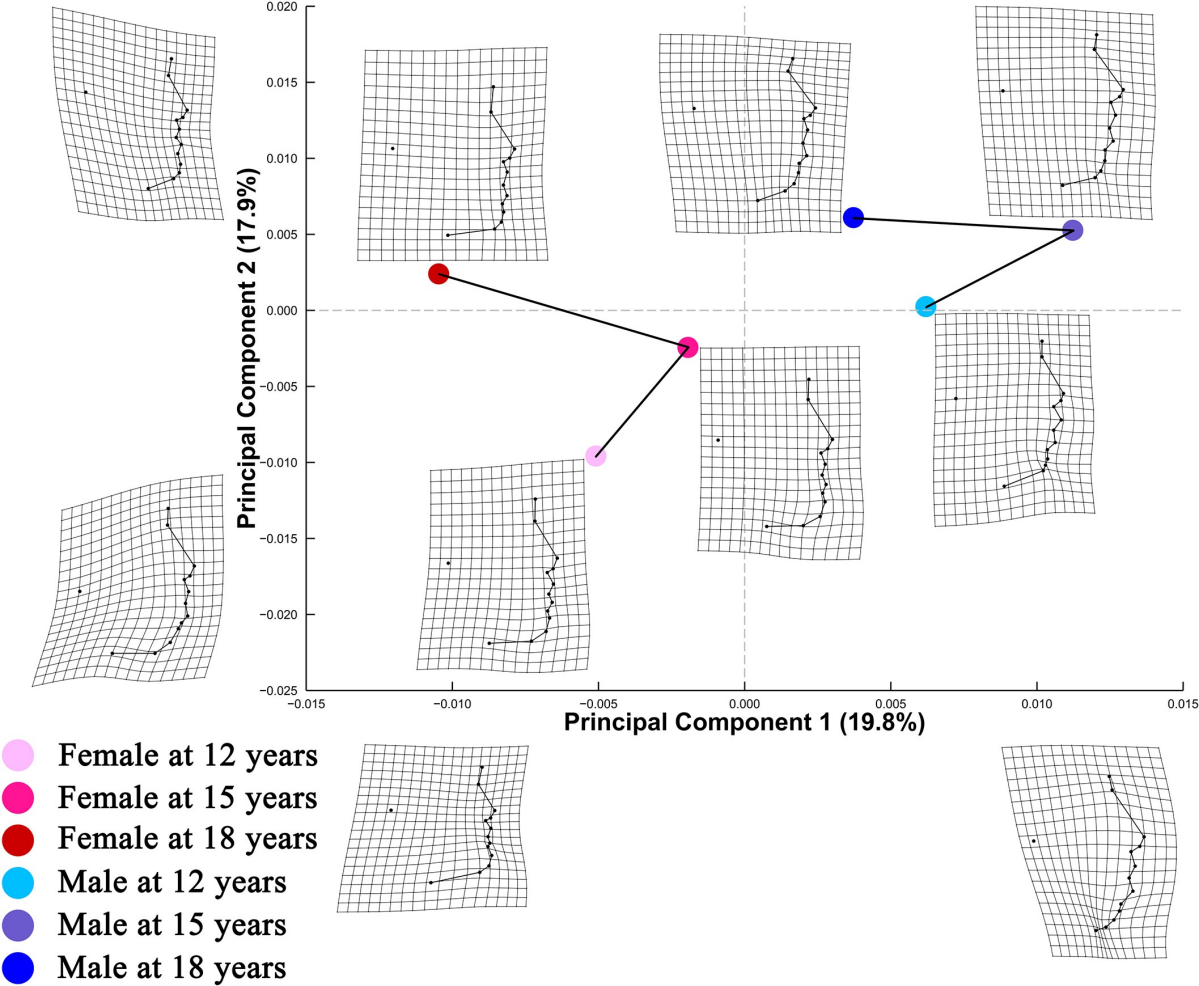
PC plot of Procrustes shape coordinates for lateral facial landmark configurations. Points represent mean PC score for the age-by-sex groups along the first two PCs. Black lines are two dimensional representations of the phenotypic growth trajectories that exist in the multidimensional shape space. The relative amount of shape variation explained by the first two PCs are shown in percentage. TPS grids along the axes illustrate shape difference between configurations with low/high PC scores and the grand mean shape, with ×2.5 magnification of the real shape difference. Mean shape for each age×sex group is illustrated relative to the grand mean shape using TPS grids next to the corresponding point, with ×4 magnification.

For lateral facial configurations (Fig 4), PC1 and PC2 explained 19.8% and 17.9% of shape variation, respectively. Shape information encoded along PC1 reflected contrasting spatial distributions of nasal, orolabial, and mentocervical landmarks. Travelling along the positive direction of PC2, there was a marked relative upward displacement of the tragion point.

Phenotypic growth trajectories along the first two PCs showed that developmental changes of facial shape did not take place in a linear fashion (Fig 4). Superimposed facial shapes (Fig 3C) revealed that for several landmarks, the direction of displacement from 15 to 18 years was opposite to the direction of displacement from 12 to 15 years.

*Developmental changes in variance of facial shape*: Variance of lateral facial configurations (Table 4) decreased 13.6% from 12 to 18 years (p = 0.0193) for females, likewise, a 12.8% reduction in variance was observed from 12 to 18 years (p = 0.0257) for males.

*Comparisons of phenotypic growth trajectories between sexes*: Table 5 revealed that the direction of developmental changes in lateral facial shape differed between sexes (*θ* = 93.1695, *p* value = 0.0406). In addition, females and males differed significantly in shapes of the phenotypic growth trajectories (*D*_*shape*_ = 0.3876, *p* value = 0.0452). In contrast, the total amount of shape change was similar between sexes (*MD* = -0.0006, *p* value = 0.9057).

*Developmental changes in sexual dimorphism of facial shape*: For lateral facial configurations, the amount of sexual dimorphism remained stable from age 12 through 15 to 18 years, as shown by the pairwise 95% confidence intervals that included the zero point (Table 6).

#### 3.4.2 Developmental shape changes after removal of allometric effect

*Multivariate regression of lateral facial shape on In(CS)*: Multivariate regression (Table 7) showed that the association between *In(CS)* and shape was significant for all age-by-sex groups (p < 0.05) except for male facial shapes at age 18 years (p = 0.0751). The percentage of shape variation attributable to size ranged from 1.50% to 6.14%.

**Table 7:** Multivariate regression of lateral facial shape on *In(CS)* for each age-by-sex group.

		Predicted shape variation (%)	p-value	
Females				
	12 years	6.14	0.0001	***
	15 years	2.24	0.0013	**
	18 years	2.73	0.0003	***
Males				
	12 years	5.82	0.0001	***
	15 years	2.18	0.0120	*
	18 years	1.50	0.0751	

***p< 0.001

**p< 0.01

*p< 0.05.

Lateral facial shape variation predicted by *In(CS)* is expressed as the percentage of total shape variation.

**Fig 5:**
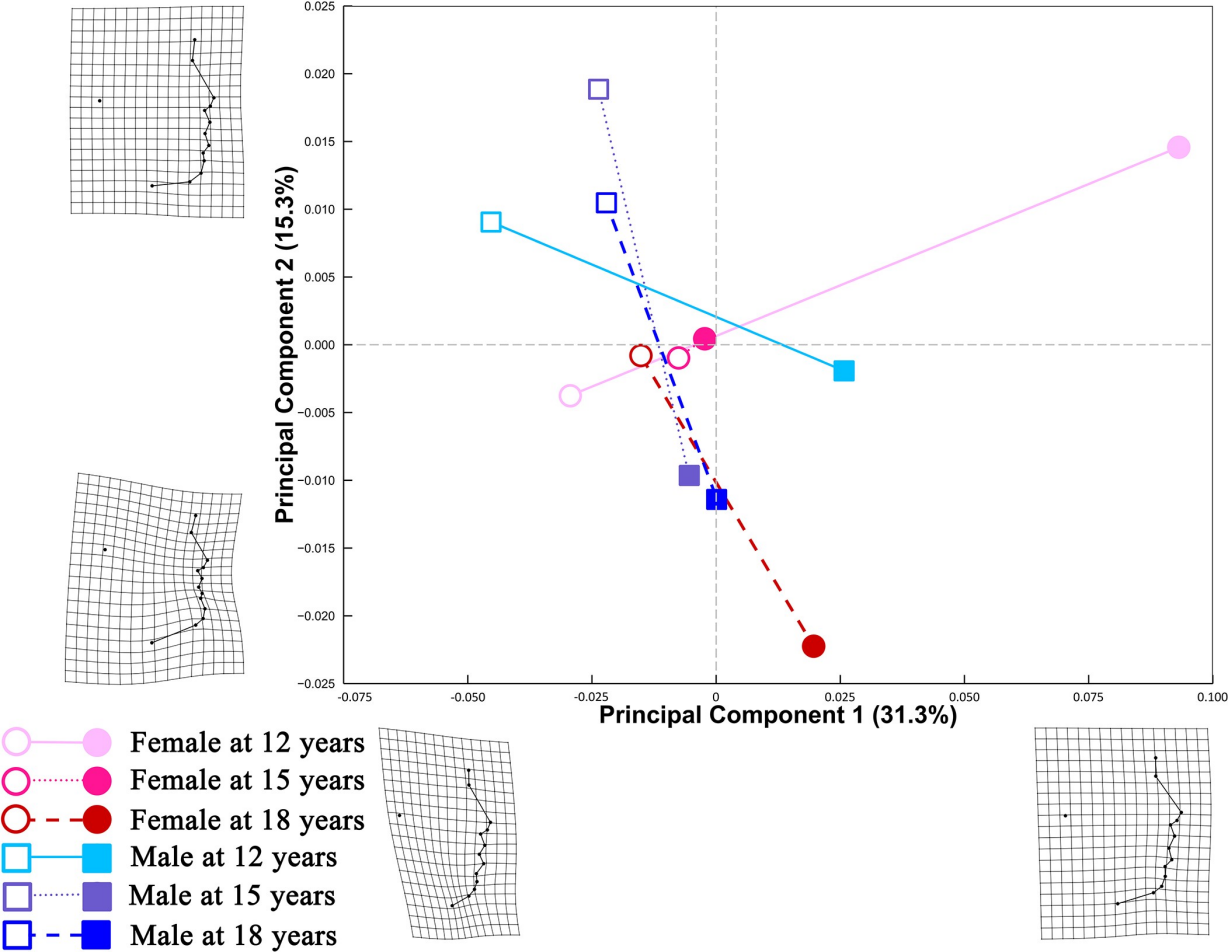
Allometric trajectories for each age-by-sex group in the first two PCs. Lateral facial shapes were estimated at the smallest and largest *In*(*CS*) of each age-by-sex group. Lines extending from the predicted mean shape at the smallest *In*(*CS*) (open symbols) to the predicted mean shape at the largest *In*(*CS*) (solid symbols) are two dimensional representations of the static allometric trajectories that exist in the multidimensional shape space. The relative amount of shape variation explained by the first two PCs are shown as percentages. TPS grids along the axes illustrate shape differences between individuals with low/high scores along either PC and the grand mean shape, with ×2.5 magnification.

A PC plot (Fig 5) comprising predicted shapes at both small and large *In(CS)* showed that PC 1 accounted for 31.3% of total shape variation, differentiating lateral facial configurations with varying degree of relative displacement of the cervical point and menton point. PC 2 explained 15.3% of shape variation, with higher PC 2 scores indicative of more sparsely arranged orolabial landmarks. Different patterns of allometry among the age-by-sex groups were suggested by the nonparallel static allometric trajectories.

**Fig 6:**
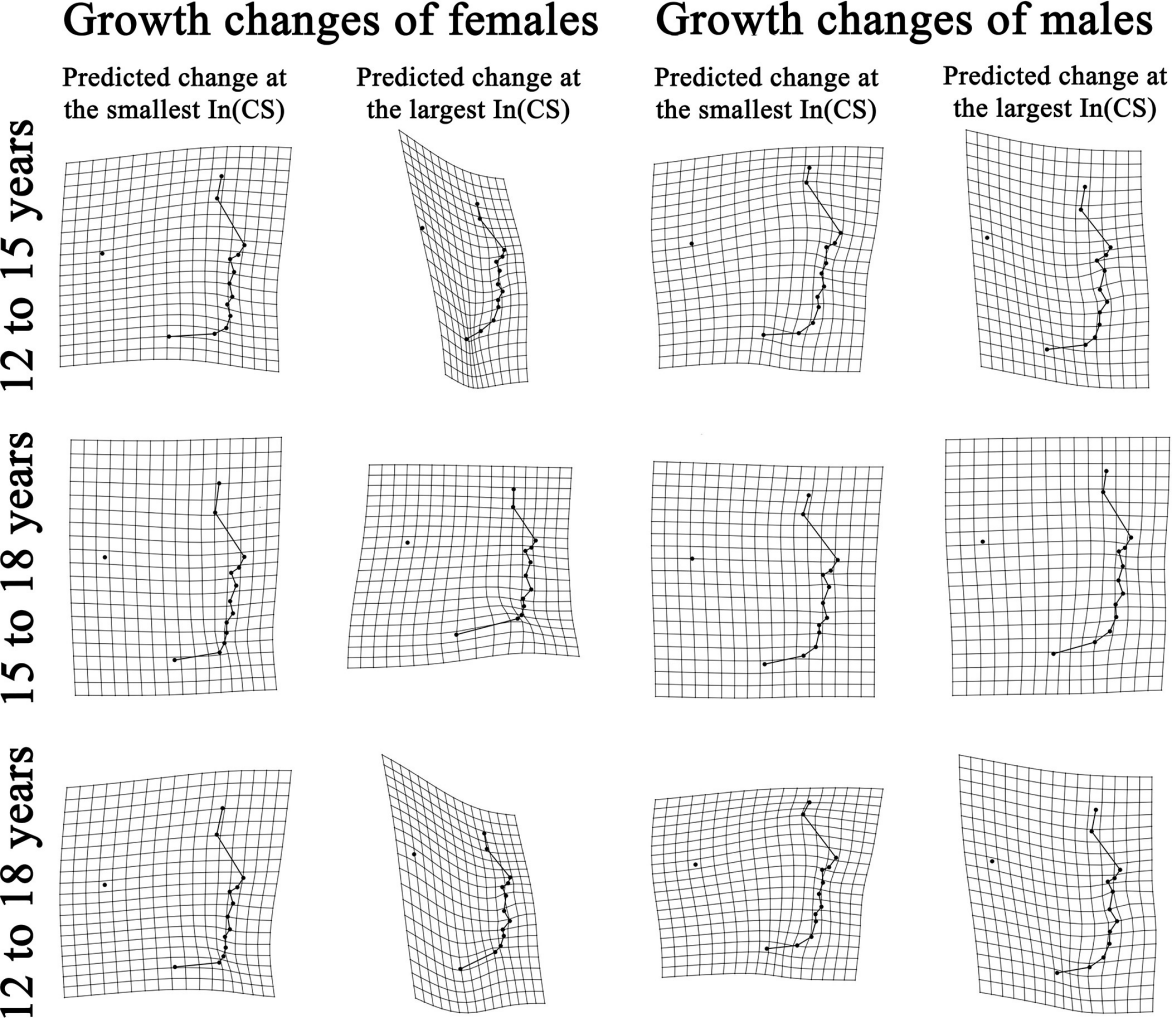
Predicted developmental shape changes of lateral facial morphology. TPS grids represented facial shape at the younger age (reference shape) warped to the facial shape at the older age (target shape), with ×3 magnification.

Visualizing developmental changes of lateral facial shape at different levels of *In(CS)*: TPS grids were used to provide direct visualization of developmental shape changes of lateral facial morphology (Fig 6). Developmental shape changes were illustrated separately for the mean shapes predicted at each age-by-sex groups’ smallest *In(CS)* and the mean shapes predicted at the group’s largest *In(CS)*.

*Rate of change in lateral facial shape with In(CS) among the age-by-sex groups*: Pairwise comparisons (Table 8) revealed that the age-by-sex groups did not differ in rate of shape change with changing *In(CS)* (p > 0.05).

**Table 8:** Pairwise differences in rate and direction of changes in lateral facial shape with changing *In(CS)* within- and between-sexes among all age-by-sex groups.

			Female						Male					
			12 years		15 years		18 years		12 years		15 years		18 years	
**Rate of shape change**														
	Females													
		12 years	-		0.9829		0.9558		0.7598		0.8360		0.5227	
		15 years	0.0011		-		0.9745		0.8477		0.8358		0.5414	
		18 years	0.0028		0.0018		-		0.8580		0.8005		0.5540	
	Males													
		12 years	0.0119		0.0108		0.0090		-		0.7001		0.6531	
		15 years	0.0128		0.0138		0.0156		0.0246		-		0.4291	
		18 years	0.0402		0.0391		0.0373		0.0283		0.0529		-	
**Direction of shape change**														
	Females													
		12 years	-		0.0002	***	0.0084	**	0.4614		0.0178	*	0.0325	*
		15 years	**90.18**		-		0.6465		0.0007	***	0.1307		0.2085	
		18 years	**67.73**		40.27		-		0.0186	*	0.4888		0.4438	
	Males													
		12 years	30.21		**85.74**		**64.39**		-		0.1139		0.2547	
		15 years	**73.27**		65.60		47.68		57.91		-		0.9980	
		18 years	**67.51**		60.11		48.91		49.87		20.51		-	

***p<0.001

**p< 0.01

*p< 0.05.

For the ‘rate of shape change’ section, values below diagonal are pairwise differences in the amount of lateral facial shape change per unit change of *In*(*CS*), measured in PD; above diagonal are *p*-values for the pairwise comparisons. For the ‘direction of shape change’ section, values below diagonal are pairwise differences in direction of the static allometric trajectory, measured in degrees; above diagonal are p-values for the pairwise comparisons. Significant directional differences are bolded.

*Direction of change in lateral facial shape with In*(*CS*) *among the age-by-sex groups*: Pairwise comparisons (Table 8) revealed that the direction of static allometric trajectory for females at age 12 years differed from the trajectory direction for both females and males at age 15 and 18 years (p < 0.05). The trajectory direction for males at 12 years differed from the direction of trajectory for females at both age 15 and 18 years (p < 0.05). In spite of the perpendicular static allometric trajectories for females at age 12 and 15 years (90.18 degrees), the two trajectories appeared parallel in the PC plot (Fig 5). Bearing in mind that the PC plot is a two-dimensional representation of the multidimensional shape variables, the perpendicular relationship between the two trajectories may be visible in dimensions other than the first two PCs.

## 4 Discussion

In this prospective, population-based geometric morphometric evaluation of developmental shape changes of facial morphology, mean facial shape changed significantly in both sexes with age. Variance of frontal facial shape decreased in females and variance of lateral facial shape decreased in both sexes. Comparisons of phenotypic growth trajectories between sexes revealed that the direction of developmental changes of facial shape and the shape of the path along which facial shape change took place were different between sexes while the total amount of shape change exhibited during the observation period was similar. In addition, the magnitude of sexual dimorphism was stable throughout the entire study period. Allometry had no substantial contribution to frontal facial shape development from 12 to 18 years. For lateral facial configurations, the age-by-sex groups differed in directions of allometric trajectories, but not in rate of shape change with changing *In*(*CS*).

### 4.1 Measurement error

Most geometric morphometric studies of soft tissue facial structures failed to report measurement error. An exception is Pound et al.’s study [32] on facial fluctuating asymmetry, where measurement error was quantified through intraclass correlation coefficients of the original landmark coordinates and Procrustes ANOVA of the Procrustes shape coordinates. Quantification of measurement error is of particular importance for studies of fluctuating asymmetry since large measurement error would confound the observed asymmetry [33]. Similarly, quantification of measurement error in longitudinal studies are necessary in that developmental changes may be masked by measurement error. Measurement error in the present study is not of serious concern due to the low percent measurement error, which is comparable with landmark reliability of a geometric morphometric study on human external ear [34]. In addition, the large sample size and longitudinal nature of this study allowed for high power to detect developmental changes in the presence of measurement error.

### 4.2 Growth changes in CS

In a longitudinal geometric morphometric analyses of skull shape [10], it was found that size of the lateral facial skeletal structures ceased to increase at age 15 among females. However, our results indicated a small but significant increase in *In*(*CS*) from 15 to 18 years among females in both frontal and lateral views. This may reflect the fact that developmental changes of soft tissue facial structures do not strictly follow changes of the underlying skeletons. In addition, the inconsistent findings may also arise from differences in the set of landmarks selected for analyses.

The *In*(*CS*) for frontal facial shape increased steadily from 12 through 15 to 18 years of age. However, for lateral facial configurations, a decrease in *In*(*CS*) was noted at age 15. The CS for lateral facial configurations reflected the size of the area enclosed by the cervical point, the tragion point, and the facial midline landmarks between glabella and menton. The reduction in *In*(*CS*) cannot be construed to mean that the size of the entire lateral facial area decreased. It may be more sensible to interpret this reduction as a reflection of the complex patterns of relative landmark displacement during growth.

### 4.3 Interaction between CS and age-by-sex groups

While declaring differences in patterns of allometry among the age-by-sex groups, coefficients of partial determination should be taken into consideration in addition to statistical significance of the interaction term. Our results for lateral facial configurations (Table 6) showed that *partial R*^2^ for the interaction term was of a similar magnitude to the main effects of *In*(*CS*) and age-by-sex groups. It was therefore reasonable to believe that the significant interaction represented genuine differences in static allometric trajectories rather than a mere artefact of the relatively large sample size in this study.

In a geometric morphometric study comparing facial shape of 19 males aged 6–11 years and 25 males aged 17–33 years, Mitteroecker et al. [9] found that allometry was not significant in the adult sample and suggested that allometry was weaker in adults than in children. In line with this, we found that static allometry was not significant in males at age 18 (Table 7). In females, the proportion of variance explained by size was smaller at age 15 and 18 than at 12 years of age.

### 4.4 Developmental shape changes of facial morphology

#### 4.4.1 Developmental changes in mean facial shape

PC plot of frontal facial configurations (Fig 1) revealed a general trend of elongation of the middle and lower facial area with growth in both sexes. Using geometric morphometric methods, similar developmental changes were noted by Mitteroecker et al. [9] through comparison of frontal facial configurations of 19 males aged 6–11 years and 25 males aged 17–33 years. In the lateral view, tragion point was found to move upward relative to structures along the facial midline in both sexes with growth. Despite these patterns of developmental changes, the amount of shape change was notably small in both frontal and lateral views, as shown by the relatively small coefficient of partial determination for the main effect of age (S2 Appendix) and the small PD between age levels (Table 3).

Alberch et al. [35] conceptualized ontogenetic trajectory of biological organisms as a curved, nonlinear line in the age-size-shape space. Consistent with Alberch’s conceptual framework, Zelditch et al. [36] found that ontogenetic trajectory of mandible shape of the mouse changed direction during ontogeny. The present study expanded the scope of previous research to human soft tissue facial structures. Our findings that the magnitude of shape change from 12 to 18 years was evidently smaller than the sum of changes from 12 to 15 years and from 15 to 18 years lend support to the hypothesized nonlinear pattern of shape ontogeny. In addition, the phenotypic growth trajectories on PC plots (Figs 1 and 4) and the directions of landmark displacement on the superimposed facial shapes (Fig 3) are also suggestive of a non-linear pattern of developmental changes in facial shape. It should be noted that the TPS grids in Figs 1, 2 and 4 captured shape variation along the two most prominent PCs but should not be taken as the real ontogenetic changes that took place during the study period.

Although the direction of developmental changes in facial shape was not consistent from age to age, phenotypic growth trajectory for males (Fig 1) failed to capture this directional change. This is because PC plot is a two-dimensional simplification of the multidimensional shape variables. Although PC plots are commonly used to facilitate visualization of major shape variations, they cannot be expected to capture all information in the multidimensional shape space.

#### 4.4.2 Developmental changes in variance of facial shape

Decreased phenotypic variance during ontogeny has been reported in various species, such as body weight of mouse [37], skull size of rats [38], barb length of the warbler [39], and size of snails [40]. Using a geometric morphometric approach, Fischer-Rousseau et al. [41] reported that variance in shape of brook charr (a species of fish) almost halved during ontogeny. In European cave salamanders, juvenile foot morphology demonstrated higher levels of variability than that of adults [25]. In line with these studies, our findings suggest that variance of facial shape decreased during development in a sample of Chinese in Hong Kong. Individual variation during shape development can be decomposed into variation in shape of ontogenetic trajectories and variation in the location of individuals along their ontogenetic trajectories, i.e., the degree of maturity [42]. During development, individuals progress along ontogenetic trajectories. As individuals reach maturity, shape variation among individuals associated with varying levels of maturity decreases [42]. This possibly explains the reduced facial shape variation observed in the present study.

It should be noted that the present study focused exclusively on the symmetric component of human face. Fluctuating asymmetry also affects shape of human face and it has been reported to increase during adolescence due to accumulation of random developmental errors over time [43]. Therefore, the observed reduction in variance of the symmetric component of human face in this study cannot be construed to mean reduced variation in the overall facial shape since the effect of fluctuating asymmetry on facial shape is not taken into consideration. Future studies investigating the relative contributions of the two components of facial shape to overall facial shape would provide insight into age-related changes in variance of facial shape.

#### 4.4.3 Comparisons of phenotypic growth trajectories between sexes

Sexual dimorphism in craniofacial features emerges early during individual development [44]. A longitudinal cephalometric study further revealed that facial features that differed between sexes during infancy were different from those that differed between sexes at adults [32]. Similarly, Wellens et al. [11] identified mild divergence in ontogenetic allometric trajectories between sexes based on lateral cephalograms. However, the path along which facial development takes place remains elusive and sexual differences in phenotypic growth trajectories have not been investigated. Our findings suggest that during adolescence, the direction of developmental changes of facial shape was different between sexes, both in frontal and lateral views. In addition, we found that the total amount of shape change was similar between sexes during the observation period while there was significant sexual dimorphism in the shape of the path along which facial shape changed.

#### 4.4.4 Developmental changes in sexual dimorphism of facial shape

Although Table 5 indicated sexually dimorphic orientation in phenotypic growth trajectories, Table 6 suggested that the magnitude of sexual dimorphism in facial shape remained stable from age 12 through 15 to 18 years. This is due to the small, albeit significant, age-related changes in facial shape within both sexes (Table 3). In spite of the differing directions of shape development, the magnitude of shape changes for both sexes along their respective trajectory is so small that it did not significantly influence the magnitude of sexual dimorphism of facial shape during the age period investigated.

### 4.5 Developmental changes in lateral facial shape taking allometry into consideration

The statistically significant interaction between *In*(*CS*) and age-by-sex groups could be visualized through the nonparallel static allometric trajectories (Fig 5). TPS grids (Fig 6) demonstrated different patterns of developmental changes of lateral facial shape for configurations with small and large CS.

In accordance with Bulygina et al. [10], our findings suggest that the rate of lateral facial shape change with changing *In*(*CS*) did not differ between sexes or among age levels (Table 8). Furthermore, we found that the significant interaction between *In*(*CS*) and age-by-sex groups was attributable to the directional differences in the static allometric trajectories (Table 8).

### 4.6 Study implication

Knowledge on dentocraniofacial [45] development has long been of interest. Understanding developmental shape changes of facial morphology may be useful in prediction of facial shape at a particular age. Compared to traditional anthropometric measurements, shape information is more comprehensive and holds potential as a new biometric personal identifier. In addition, developmental facial shape changes in healthy populations may be compared with changes in patients with craniofacial abnormalities. Such comparisons provide an opportunity to deepen our understanding of developmental process of the diseases.

### 4.7 Strengths and limitations

To the best of our knowledge, this is the first longitudinal geometric morphometric investigation of developmental shape changes of human soft tissue facial structures. The large sample size and longitudinal nature of this study enabled us to detect developmental changes of the face with high power. To provide an in-depth understanding of developmental changes of facial shape, various aspects of shape changes were analyzed through advanced statistical methods initially developed for other research fields. For example, the phenotypic growth trajectory analysis was developed to study phenotypic variation across evolutionary levels [23]. In addition, the method for analyses of developmental changes of shape difference between sexes was used by developmental biologists to study ontogenetic convergence and divergence [28]. Taking allometry into consideration in our analyses provided us an in-depth understanding of developmental shape changes of facial morphology. Furthermore, the morphometric procedures outlined in this study may serve as a methodological reference for future studies.

This study also has limitations that need to be addressed. First, the graphs on which facial shapes were superimposed (Fig 3) were not directly comparable with superimposed tracings of cephalograms in orthodontic literature [46, 47]. This is because the process of GPA removed size information from landmark coordinates and thus the resultant graphs do not reflect the absolute amount of developmental changes represented by the cephalometric tracings. In addition, landmark configurations were rotated iteratively during GPA to minimize the average PD. Therefore, unlike superimposition of cephalograms [48], there was no pre-determined reference system during GPA. Second, within the limitations of two-dimensional photogrammetry, this study revealed different patterns of interaction between *In*(*CS*) and age-by-sex groups in frontal and lateral views. It would be of interest to examine this interaction through landmark data obtained from three-dimensional photogrammetric techniques so that the entire constellation of facial landmarks could be analyzed and visualized simultaneously. In addition, due to sparsity of anatomical landmarks defining the upper third of the face, analyses in the present study were limited to lower two thirds of the face. Algorithms for automatic digitization of spatially-dense three-dimensional semilandmarks covering the entire human face have been developed [49]. Future geometric morphometric studies based on three-dimensional facial photographs and advanced algorithms for landmark digitization would allow for increased accuracy and complete coverage of the human face. Third, CS explained only a small proportion of facial shape variation. The effect of other variables in addition to CS on facial shape has also been investigated in geometric morphometric studies. Theses variables could also be taken into consideration in future analyses of changes of facial shape. Fourth, age of the participants was restricted to 12 to18 years, a period that possibly excluded the onset of pubertal facial skeletal growth for girls. Increasing the investigative period to age 10 years will allow for a more complete understanding of developmental changes of facial shape during adolescence. However, pubertal facial skeletal growth peaks at age 14 and 12.5–13 years for boys and girls [50], respectively, which were included within the investigative period of this study, hence we were able to capture the most important pubertal changes in facial shape for both sexes.

## 5 Conclusions

In summary, we observed small but significant changes in mean and variance of facial shape in both sexes with growth. Direction and shape of phenotypic growth trajectories differed between sexes, however, the magnitude of sexual dimorphism remained stable during the observation period. Allometry minimally influenced developmental shape changes of frontal facial morphology. For lateral facial configurations, patterns of allometry differed among age levels and between sexes. These differences were attributable to differences in direction rather than rate of the allometric trajectories.

## Supporting information

S1 Appendix Definitions of the anthropometric landmarks used. (DOCX)

S2 Appendix Main effect of age, sex, and their interaction from permutational MANOVA. ***p< 0.001. Partial *R*^2^ = coefficient of partial determination.
(DOCX)

S1 Fig Schematic illustrations of landmarks digitized on frontal face. This figure is adapted from one of our previous publications [12].
(TIF)

S2 Fig Schematic illustrations of landmarks digitized on lateral face. This figure is adapted from one of our previous publications [12].
(TIF)

S3 Fig Distributions of *In*(*CS*) by sex and age. The white horizontal bar inside the box represents mean *In*(*CS*). (A) *In*(*CS*) for female frontal facial configurations; (B) *In*(*CS*) for male frontal facial configurations; (C) *In*(*CS*) for female lateral facial configurations; (D) *In*(*CS*) for male lateral facial configurations.
(TIF)

S1 Data Coordinates for frontal facial images of females. (TXT)

S2 Data Coordinates for frontal facial images of males. (TXT)

S3 Data Coordinates for lateral facial images of females. (TXT)

S4 Data Coordinates for lateral facial images of males. (TXT)
