# Supplementary material for: Developmental shape changes in facial morphology: Geometric morphometric analyses based on a prospective, population-based, Chinese cohort in Hong Kong
Source: PLoS One. 2019 Jun 28;14(6):e0218542. doi: 10.1371/journal.pone.0218542 (PMC6599092; doi:10.1371/journal.pone.0218542)
Supplement: S2 Appendix — ***p< 0.001. Partial R2 = coefficient of partial determination. (DOCX) [file pone.0218542.s002.docx]

**Supplementary Table 2.** Main effect of age, sex, and their interaction from permutational MANOVA.

| MANOVA Model | Frontal images | | |  | Lateral images | | |
| --- | --- | --- | --- | --- | --- | --- | --- |
|  | partial $R^{2}$ | $p$-value | |  | partial $R^{2}$ | $p$-value | |
| age | 0.0378 | 0.0001 | *** |  | 0.0147 | 0.0001 | *** |
| sex | 0.0314 | 0.0001 | *** |  | 0.0329 | 0.0001 | *** |
| age×sex | 0.0078 | 0.0003 | *** |  | 0.0085 | 0.0001 | *** |

***$p<$ 0.001. Partial $R^{2}=$ coefficient of partial determination.
