# Supplementary figures and images for: Developmental shape changes in facial morphology: Geometric morphometric analyses based on a prospective, population-based, Chinese cohort in Hong Kong
Source: PLoS One. 2019 Jun 28;14(6):e0218542. doi: 10.1371/journal.pone.0218542 (PMC6599092; doi:10.1371/journal.pone.0218542)

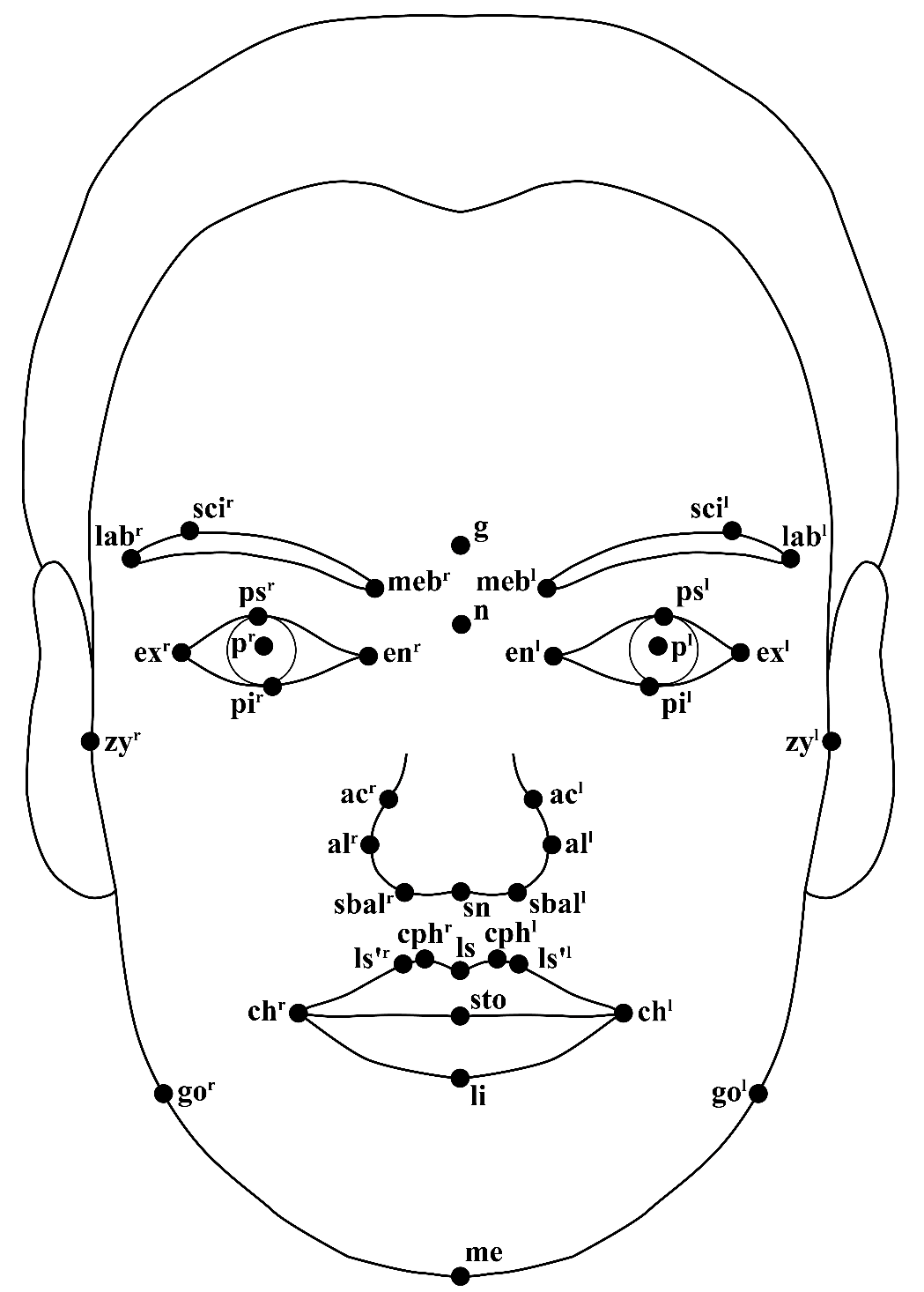

Supplement: S1 Fig — This figure is adapted from one of our previous publications [12]. (TIF) [file pone.0218542.s003.tif]

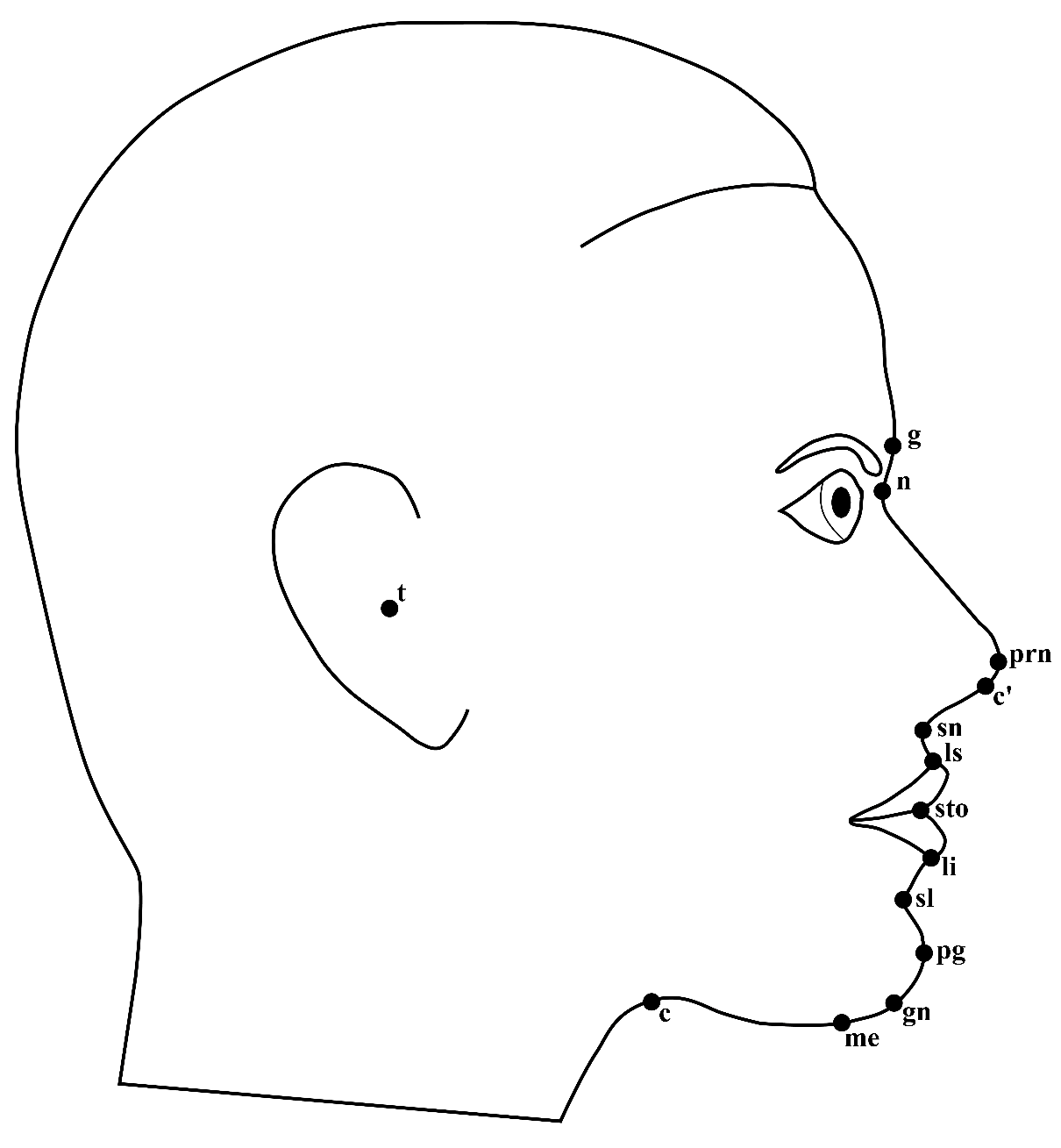

Supplement: S2 Fig — This figure is adapted from one of our previous publications [12]. (TIF) [file pone.0218542.s004.tif]

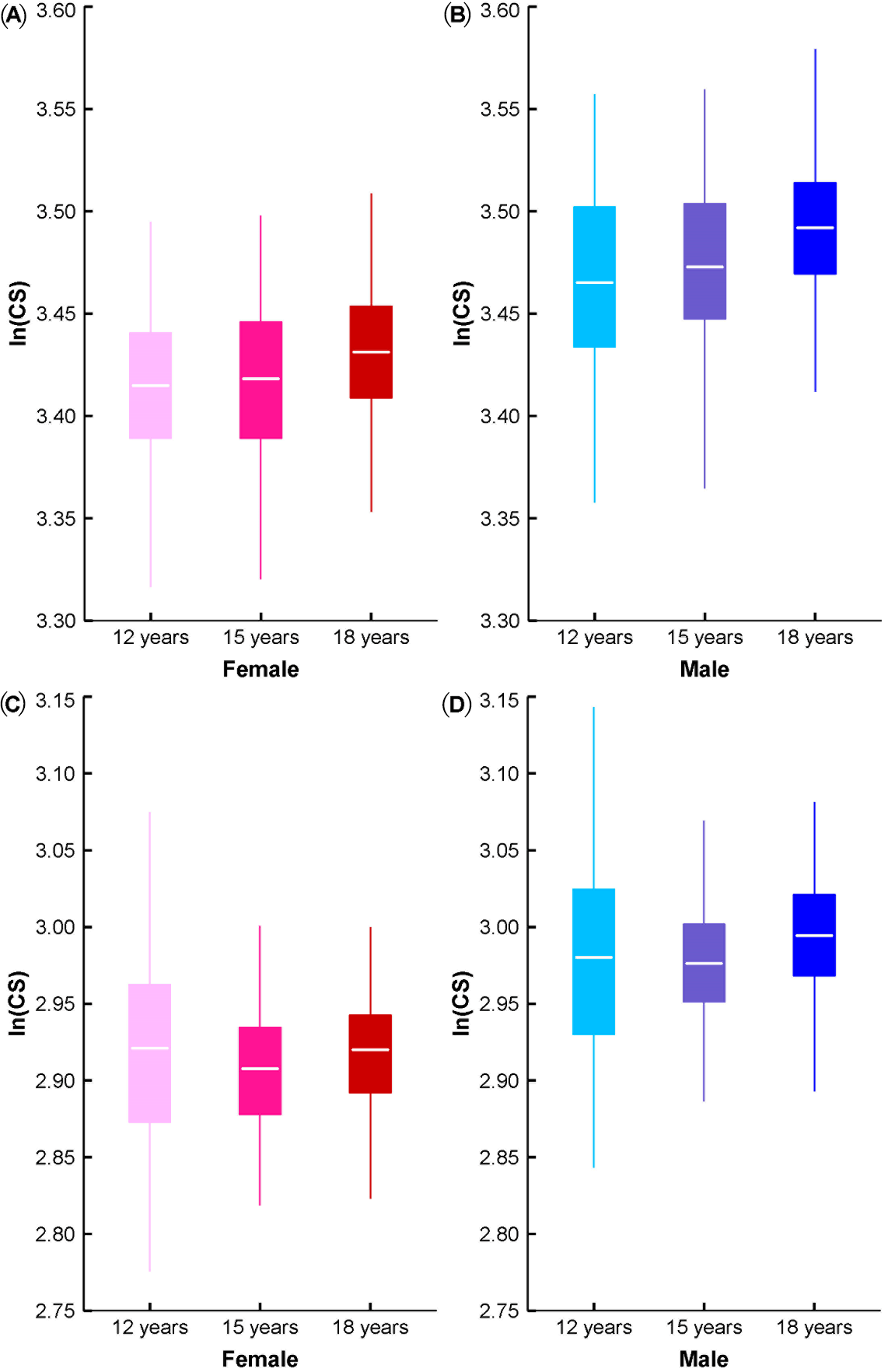

Supplement: S3 Fig — The white horizontal bar inside the box represents mean In(CS). (A) In(CS) for female frontal facial configurations; (B) In(CS) for male frontal facial configurations; (C) In(CS) for female lateral facial configurations; (D) In(CS) for male lateral facial configurations. (TIF) [file pone.0218542.s005.tif]
